# High Performance Liquid Chromatography Coupled with Pre-column Derivatization for Determination of Oxidized Glutathione Level in Rats Exposed to Paraquat 

**Published:** 2013

**Authors:** Zahra Hami, Mohsen Amini, Amir Kiani, Mahmoud Ghazi-Khansari

**Affiliations:** a *Department of Pharmacology, School of Medicine, Tehran University of Medical Sciences, Tehran, Iran. *; b *Department of Medical Nanotechnology, School of Advanced Technologies in Medicine, Tehran University of Medical Sciences, Tehran, Iran. *; c *Department of Medicinal Chemistry, Faculty of Pharmacy and Drug Design and Development Research Center, University of Medical Sciences, Tehran, Iran. *; d *Department of Pharmacology, Toxicology and Medical Services, School of Pharmacy, Kermanshah University of Medical Sciences, Kermanshah, Iran. *

**Keywords:** Glutathione disulfide (GSSG), Paraquat (PQ), Oxidative stress, 9-fluorenylmethylchloroformate (FMOC), HPLC

## Abstract

Glutathione (GSH) is one of the most important antioxidants that plays an essential role in detoxification of reactive oxygen species (ROS) which oxidizes to glutathione disulfide (GSSG). Paraquat (PQ), awidely used herbicide*, *causes pulmonary injury with the productionof ROS. Excessive ROS accumulation as a consequence of PQ exposure are frequently targeted by GSH thereby oxidative stress leads to depletion of cellular GSH by transforming of GSH to glutathione disulfide (GSSG). A precise method of measuring of GSSG concentration in plasma as indicator of oxidative stress is needed. Some analytical techniques such as high-performance liquid chromatography (HPLC), gas chromatography and capillary electrophoresis have been used for determination of GSSG concentration. In the present study, a new HPLC method with fluorescence detection based on derivatization of the amine group of glutathione with 9-fluorenylmethyl chloroformate (FMOC-Cl) was developed. Male Wistar albino rats exposed to different doses of PQ (20-60 mg/kg) and control group were used and after protein precipitation, their plasma was subjected to derivatization with FMOC in the presence of borate buffer. The derivatized samples were injected to HPLC system with C18 column, mobile phase consisting of methanol and phosphate buffer, λ_em_= 315 nm, λ_ex_= 260 nm. Among all experimental groups, the rats which received 60 mg/kg PQ, showed a significant increase in the amount of oxidized glutathione (GSSG) compared to the control group. In this study, the applied derivatization and HPLC method made it possible to measure small amounts of glutathione in plasma using a precise and sensitive technique.

## Introduction

Paraquat (PQ; 1,1’-dimethyl-4,4’-bipyridylium dichloride) is one of the most used herbicides that induces oxidative stress (1). It has been shown that NADPH-cytochrome P450 reductase can produce paraquat radical by one electron reduction of paraquat. In the presence of oxygen, paraquat radical would be auto oxidized and leads to production of paraquatdication and reactive oxygen species (ROS) such as hydrogen peroxide, super oxide and hydroxyl radicals (2-5). 

These toxic metabolites, will be reduced by sequential function of two protective enzymes,glutathione peroxidase (GPX) and glutathione reductase (GR), in their redox cycle. In this process, glutathione (GSH) will oxidize to glutathione disulfide (GSSG) (6-9). Glutathione (*γ*-glutamylcysteinylglycine) is a unique tripeptidethiol in most plants, microorganisms and all mammalian tissues (10). Glutathione high reducing ability is responsible for cell protection against oxidative damages (11). Therefore, oxidative stress leads to depletion of cellular GSH by transforming of GSH to GSSG. GSSG concentration in plasma is very low under physiological conditions, so measuring the increasing amount of GSSG is a sensitive tool for evaluation of redox states (12). Blood glutathione concentrations can reflect glutathione tissue levels which are not accessible or less accessible; therefore measuring blood glutathione is a good index for pathological evaluation of many diseases. Many methods such as enzymatic techniques (13), high-performance liquid chromatography (HPLC) (14), gas chromatography (15), nuclear magnetic resonance (NMR) spectroscopy (16) and capillary electrophoresis (17) have been used for measuring glutathione in biological samples.

Enzymatic techniques don’t need advanced equipment but they don’t have a low enough detection limit and often have a low reproducibility. These methods are time- consuming and laborious, so they are not useful for routine analysis.

NMR and capillary electrophoresis are used less, as they are expensive. Among the above mentioned methods, HPLC with fluorescence detector is a precise, sensitive, and selective method and is suitable for measuring glutathione in samples such as plasma with low amount of GSSG. FMOC is a fluorescent derivatizing agent that reacts with primary and secondary amino groups. FMOC has been used for the derivatization of polyamines (18), aminopolyphosphonates (19), glucosamine (20) and amino acids (21).

In this study, feasibility of derivatization of glutathione with FMOC as a fluorescence derivatizing agent and measuring the resulted derivatizedanalyte with HPLC were studied.

## Experimental

*Chemicals*

Paraquat dichloride salt, 9-fluorenylmethyl chloroformate (FMOC) and heparin were purchased from the Sigma Chemical Co. (St. Louis, Mo). Acetonitrile, reduced glutathione, phosphoric acid and methanol were obtained from Merck (Germany). Triethylamine was obtained from Riedel-de Haën, Boric acid was purchased from Panreac (Spain) and potassium hydroxide was obtained from Carl Roth (Karlsruhe, Germany).

*Animals*

Thirty male Wistar albino rats (200-250 g), from the animal colony of Tehran University of Medical Sciences, department of pharmacology were used. The animals were maintained in single cages at an ambient temperature of 22–25°C at humidity of 50%–60%, with a 12 h light/dark cycle with access to standard food pellet diet and drinking water ad libitum. All the ethical issues as proposed by the Tehran University of Medical Sciences Animal Ethical Committee were strictly followed during the maintenance of the animals as well as killing the animals. The rats divided into 5 groups of 6 animals per each group. One group considered as a control (received water) and the other groups received different doses of paraquat (20, 30, 40 and 60 mg/kg,intraperitoneally; IP), respectively.

*Sample preparation and derivatization procedure*

PQ dissolved in deionized water and was injected intraperitoneally (IP) (22) at doses of 20, 30, 40, 60 mg/kg and after 4 h blood samples were collected. Rats were anesthetized with ether and then 2 mL of blood was collected with a syringe coated with heparin from rat heart ventricle and blood samples were transferred into tubes containing heparin.Tubes containing blood samples were centrifuged immediately at 4000 rpm for 15 min. Then 100 μL of supernatant (plasma) was transferred to the other tube and mixed with 300 μL of acetonitrile for precipitation of plasma proteins. After vortexing, tubes were centrifuged at 5000 rpm for 10 min. Resulted supernatant was dried in a water bath at 40°C under air flow due to air pump. Dried supernatant were mixed with 50 μL tap water, 50 μL borate buffer (0.5 M, pH 9) and 100 μL of 500 mg/mL FMOC and kept in room temperature for 15 min for completion derivatization process. Stock solution of glutathione were prepared by dissolving of 10 mg glutathione in 10 mL tap water to obtain a concentration of 1 mg/mL. Standard solutions were prepared by serial dilutions of stock solution in distilled water. Serial dilutions of this solution were prepared. 

Derivatization was started by adding 50 μL borate buffer (0.5 M, pH 9) and 100 μL of 500 mg/mL FMOC to 50 μL of each of the standard solutions in room temperature for 15 min, therefore, the final concentrations of solutions were 0.813, 0.407, 0.203, 0.102 and 0.025 μmol/mL, respectively. 20 μL of these derivatized glutathione solutions and plasma samples were injected to HPLC system with mobile phase consisting of methanol – phosphate buffer with volume ratio of 40:60, column C18 (ODS-3 5 μm 250 × 4.6 mm), isocratic elution system at a flow rate of 2 mL/min, and GSSG was detected using excitation and emission wavelengths of 260 and 315 nm.

*Statistical analysis*

All results are expressed as mean ± SEM .For comparison between the experimental groups one-way ANOVA followed by Tukey-Kramer multiple comparison test were used. P-values < 0.05 were considered statistically significant. 

## Results

**Figure 1:**
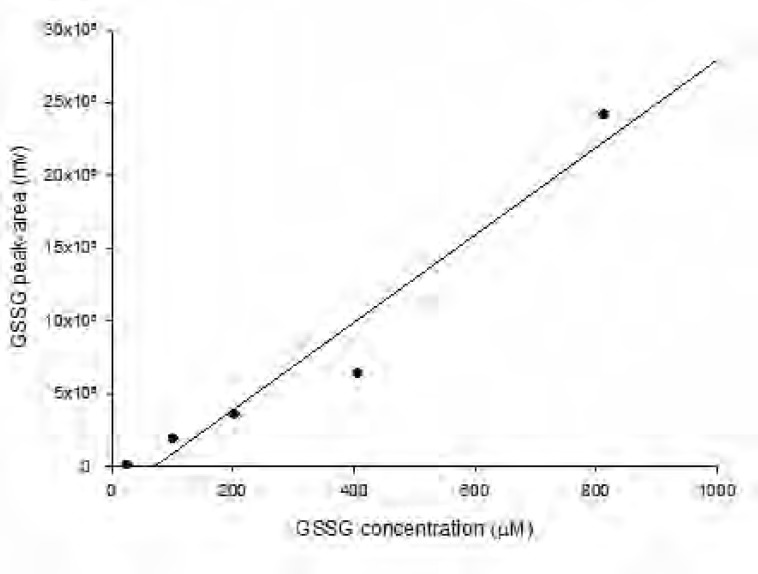
HPLC chromatogram of FMOC and its related compound, FMOC-OH in borate buffer (FMOC reacts with water to yield FMOC-OH as a hydrolysis product).

**Figure 2:**
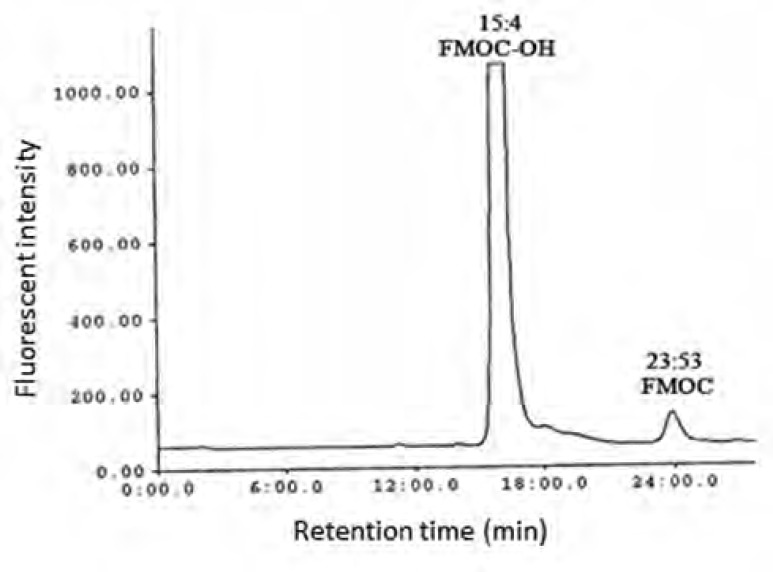
HPLC chromatogram of FMOC and its related compound, FMOC-OH in borate buffer (FMOC reacts with water to yield FMOC-OH as a hydrolysis product).

**Figure 3:**
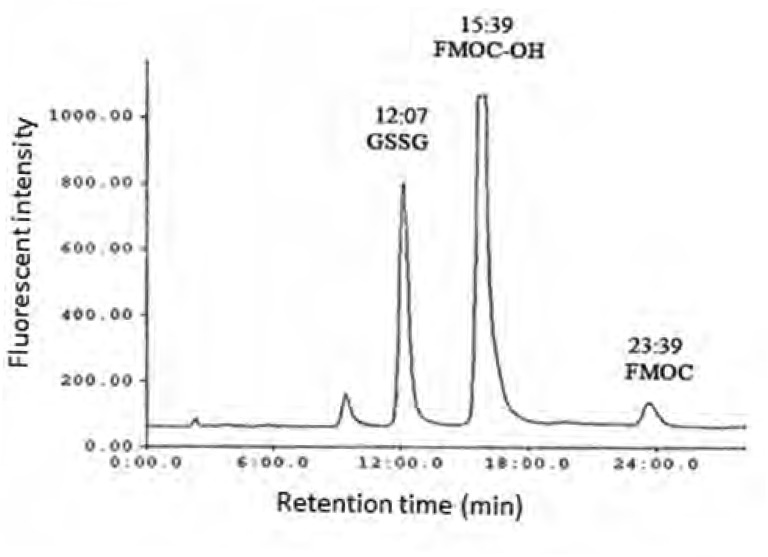
HPLC chromatogram of GSSG fluorescent derivative in borate buffer ( standard solution of glutathione in water was derivatized with FMOC in presence of borate buffer, pH 9).

**Figure 4:**
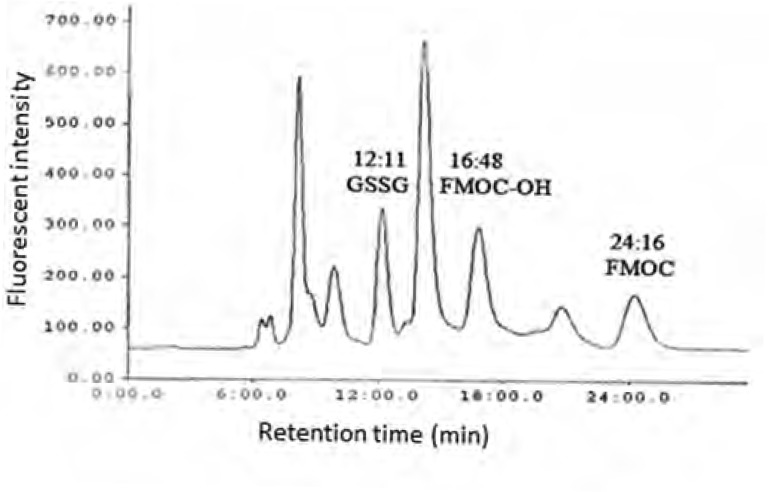
HPLC chromatogram of GSSG fluorescent derivative in rat plasma ( after exposure of rats with paraquat and derivatization of plasma GSSG with FMOC **).**

As shown in Figure 1 the amount of total glutathione is equal to GSSG because GSH has totally reduced to GSSG (r^2^ = x). Figure 2 shows chromatogram of blank solution (borate buffer + FMOC); Figure 3 shows chromatogram of standard glutathione solution (glutathione + borate buffer + FMOC) and Figure 4 shows chromatogram of plasma sample (plasma + borate buffer + FMOC).

*Paraquat dose response*

**Figure 5:**
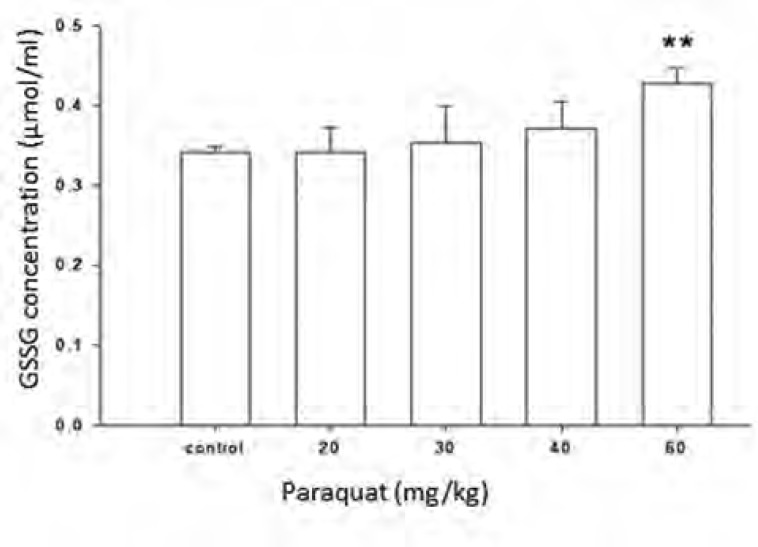
Effect of different doses of paraquat on the oxidized glutathione quantity of rat plasma. All data are given as mean ± SEM; n = 6. ** Significantly different when compared with control

Figure 5 shows the increase in the amount of total glutathione in the plasma of control rats (received water) and rats exposed to different doses of paraquat (20-60 mg/kg). The amount of total glutathione in the group exposed to 60 mg/kg of paraquat is significantly increased compared to control group. Limit of detection (LOD) and limit of quantification (LOQ) values for GSSG was 15.26 and 25.42 μM, respectively.

## Discussion

Measurement of low concentration of oxidized glutathione in plasma after oxidative stress conditions such as exposure to paraquat requires a sensitive and reliable method with low detection limit. Various methods of detection have been used (23) and in this study glutathione derivatization method with FMOC is used for derivatization of primary and secondary amines and functional groups such as -OH, -SH. Glutathione in its structure has functional groups of –SH and -NH_2_ which are capable of derivatization with FMOC. In this process thiol groups (-SH) would be oxidized to disulfide (-S-S) very fast, so using a reducing substance such as sodium borohydride (NaBH_4_) before derivatization of thiol group is essential. Moreover a solution of 4 mM of EDTA was used in order to remove interfering ions such as Ca^2+^, Mg^2+^, Na^+ ^and Fe^2+^ which facilitate the oxidation process of thiol group to disulfide. 10% trichloroacetic acid (TCA) solution was also used in order to precipitate the plasma proteins. In this study acetonitrile instead of TCA was used for precipitation of plasma proteins. Different acetonitrile volumes were used for one plasma volume and after vortexing and centrifuging for precipitation of plasma proteins, supernatant was transferred to the capped vials. 25 μL of NaBH_4_ solution in NaOH was added to 100 μL supernatant and mixed in 50°C for 0.5 h. In this procedure, both thiol group and amine group of glutathione reacted with FMOC. But as thiol group is very susceptible to oxidation (24), in spite of using reducing factor, intensity of glutathione peaks did not show this process, so thiol group was removed and just derivatization with amine group was done in order to create a peak with known molecular characterization by derivatized glutathione. For removing thiol group, glutathione solution was prepared in tap water which contains various ions such as Ca^2+^. 

These ions would change -SH group to disulfide. Thiol group is very susceptible to oxidation and can be oxidized in room temperature during several minutes, so GSH would change to GSSG immediately. In order to optimize condition of derivatization various pH ranges from 4 to 10 were applied. The results showed that acidic and neutral pH is not suitable for derivatization with FMOC so triethylamine was used for alkalinizing reaction medium. Triethylamine solution in acetonitrile in various volumes (10-50 μL) was added to reaction medium, but chromatograms did not have suitable repeatability and linear trend according to various glutathione concentrations. Finally, borate buffer with alkaline pH were used for alkalinizing. Borate buffer with pH of 8, 8.5, 9, 9.5 and 10 were prepared for evaluation of the best alkaline pH. Resulted chromatograms showed that glutathione peak in pH = 9 has more intensity. Therefore, pH = 9 was used to follow the process. Temperature factor was another variant which was evaluated for derivatization improvement. For this purpose, derivatization was done in temperatures of 40, 60, 70, 80^o^C and room temperature. It was observed that derivatization with FMOC in room temperature has the best result because derivatization in high temperatures makes lots of peaks in chromatogram which probably resulted from FMOC decomposition. For determining optimized time for derivatization, various times from 5 to 45 min were considered. It was showed that derivatization can be done during 15 min in room temperature. Mobile phase was a mixture of methanol and phosphate buffer. This buffer was prepared by solving 3 mL of triethylamine in 1000 mL of water and then the pH adjusted to 3.2 with phosphoric acid. In addition in isocratic elution with mobile phase flow rate 2 mL/min were used and then various ratios of mobile phase were evaluated. First, 75% of methanol and 25% of phosphate buffer were used. Then, ratios 15:85, 20:80, 73:27, 86:14, 12:88, 83:17 were evaluated in which higher percentage was related to methanol and lower percentage was related to buffer. None of these ratios could separate the FMOC derivative of GSSG from FMOC well. In mentioned conditions, FMOC related peaks involved FMOC peak and FMOC -OH peak (resulted from reaction of FMOC with water) were overlapped with derivatized glutathione peak and these peaks formed a crowded area in 8-10 min range. Volume ratio 40 : 60 was used for better separation which included 60% methanol and 40% phosphate buffer. In this condition, derivatized GSSG related peak was separated from FMOC peaks quietly and because of greater polarity, left the column sooner and placed in suitable space from FMOC peaks in chromatogram. In this situation, glutathione peak retention time was about 12 min, retention times of peak FMOC - OH and FMOC were around 16 and 24 min respectively. As FMOC is a suitable reagent for primary and secondary amines derivatization (25), so in this study we removed thiol group derivatization and just amine group derivatization was done. Therefore, what was derivatized and was separated and detected by HPLC was GSSG.

Paraquat is an oxidative stress inducer factor and causes oxidizing GSH to GSSG (26-27). Therefore, measurement of increase in the amount of GSSG, which in normal condition is very low rather than GSH, is a sensitive tool for measurement and evaluation of redox state. In this study, feasibility of derivatization reaction of amine group of glutathione with FMOC was evaluated for measurement of changes in the amount of glutathione due to paraquat in rat plasma by HPLC method. Figure 2 shows the effect of 4 dose paraquat on increase amount of GSSG. As shown in this diagram, just at the dose 60 mg/kg, there was a significant difference with control group and the other groups have shown a small increase in the amount of GSSG compare to control group. This study, offered a semi quantity evaluation of GSSG changes resulted from paraquat and more researches are needed in order to obtain results with higher quality and quantity, to optimize derivatization condition and also optimization of chromatography method.

## References

[1] Ghazi-Khansari M, Mohammadi-Bardbori A (2007). Captopril ameliorates toxicity induced by paraquat in mitochondria isolated from the rat liver. Toxicol. in-vitro.

[2] Somayajulu-Niţu M, Sandhu JK, Cohen J, Sikorska M, Sridhar T, Matei A, Borowy-Borowski H, Pandey S (2009). Paraquat induces oxidative stress, neuronal loss in substantianigra region and Parkinsonism in adult rats: Neuroprotection and amelioration of symptoms by water-soluble formulation of Coenzyme Q10. BMC Neurosci.

[3] Castello PR, Drechsel DA, Patel M (2007). Mitochondria are a major source of paraquat-induced reactive oxygen species production in the brain. J. Biol. Chem.

[4] Takizawa M, Komori K, Tampo Y, Yonaha M (2007). Paraquat-induced oxidative stress and dysfunction of cellular redox systems including antioxidative defense enzymes glutathione peroxidase and thioredoxinreductase. Toxicol . in-vitro.

[5] Mussi MA, Calcaterra NB (2010). Paraquat-induced oxidative stress response during amphibian early embryonic development. Comp. Biochem. Physiol. C. Toxicol. Pharmacol.

[6] Li S, Yan T, Yang JQ, Oberley TD, Oberley LW (2000). The role of cellular glutathione peroxidase redox regulation in the suppression of tumor cell growth by manganese superoxide dismutase. Cancer Res.

[7] Lu SC (2001). Regulation of glutathione synthesis. Curr. Top. Cell Regul.

[8] Griffith OW (1999). Biologic and pharmacologic regulation of mammalian glutathione synthesis. Free Radic. Biol. Med.

[9] Nasiri Bezenjani S, Pouraboli I, Malekpour Afshar R, Mohammadi G (2012). Hepatoprotective effect of Otostegia persica Boiss. shoot extract on carbon tetrachloride-induced acute liver damage in rats. Iran. Iran. J. Pharm. Res.

[10] Marchand S, De Revel GA (2010). HPLC fluorescence-based method for glutathione derivatives quantification in must and wine. Anal. Chim. Acta.

[11] Foyer CH, Noctor G (2005). Redox homeostasis and antioxidant signaling: a metabolic interface between stress perception and physiological responses. Plant Cell.

[12] Loughlin AF, Skiles GL, Alberts DW, Schaefer WH (2001). An ion exchange liquid chromatography/mass spectrometry method for the determination of reduced and oxidized glutathione and glutathione conjugates in hepatocytes. J. Pharm. Biomed. Anal.

[13] Hiraku Y, Murata M, Kawanishi S (2002). Determination of intracellular glutathione and thiols by high performance liquid chromatography with a gold electrode at the femtomole level: comparison with a spectroscopic assay. Biochim. Biophys. Acta.

[14] Abukhalaf IK, Silvestrov NA, Menter JM, von Deutsch DA, Bayorh MA, Socci RR, Ganafa AA (2002). High performance liquid chromatographic assay for the quantitation of total glutathione in plasma. J. Pharm. Biomed. Anal.

[15] Küster A, Tea I, Sweeten S, Rozé JC, Robins RJ, Darmaun D (2008). Simultaneous determination of glutathione and cysteine concentrations and 2 H enrichments in microvolumes of neonatal blood using gas chromatography–mass spectrometry. Anal. Bioanal. Chem.

[16] Potapenko DI, Bagryanskaya EG, Grigoriev IA, Maksimov AM, Reznikov VA, Platonov VE, Clanton TL, Khramtsov VV (2005). Quantitative determination of SH groups using 19F NMR spectroscopy and disulfide of 2,3,5,6-tetrafluoro-4-mercaptobenzoic acid. Magn. Reson. Chem.

[17] Yang Q, Krautmacher C, Schilling D, Pittelkow MR, Naylor S (2002). Simultaneous analysis of oxidized and reduced glutathione in cell extracts by capillary zone electrophoresis. Biomed.Chrom.

[18] Tandy S, Schulin R, Suter M, Nowack B (2005). Determination of [S,S’]-ethylenediaminedisuccinic acid (EDDS) by high performance liquid chromatography after derivatization with FMOC. J. Chromatogr. A.

[19] Nowack B (2002). Determination of phosphonic acid breakdown products by high-performance liquid chromatography after derivatization. J. Chromatogr. A.

[20] Huang TM, Deng CH, Chen NZ, Liu Z, Duan GL (2006). High performance liquid chromatography for the determination of glucosamine sulfate in human plasma after derivatization with 9-fluorenylmethyl chloroformate. J. Sep. Sci.

[21] L´opez-Cervantes J, S´anchez-Machado DI, Rosas-Rodr´ıguez JA (2006). Analysis of free amino acids in fermented shrimp waste by high-performance liquid chromatography. J. Chromatogr. A.

[22] Ghazi Khansari M, Mohammadi Karakani A, Sotoudeh M, Mokhtary P, Pour Esmaeil E, Maghsoud S (2007). Antifibrotic effect of captopril and enalapril on paraquat induced lung fibrosis in rats. J. Appl. Toxicol.

[23] Ekegren T, Gomes-Trolin C (2005). Determination of polyamines in human tissues by precolumnderivatization with 9-Xuorenylmethyl chloroformate and high-performance liquid chromatography. Anal. Biochem.

[24] Dincer Y, Akcay T, Alademir Z, Ilkova H (2002). Effect of oxidative stress on glutathione pathway in red blood cells from patients with insulin-dependent diabetes mellitus. Metabolism.

[25] Puente B, Garcia MA, Hernandez E, Bregante MA, Perez S, Pablo L, Prieto E (2011). Determination of memantine in plasma and vitreous humour by HPLC with precolumn derivatization and fluorescence detection. J. Chromatogr. Sci.

[26] Jones DP (2006). Disruption of mitochondrial redox circuitry in oxidative stress. Chem. Biol. Interact.

[27] Daraei B, Pourahmad J, Hamidi-Pour N, Hosseini MJ, Shaki F, Soleimani M (2012). Uranyl acetate induces oxidative stress and mitochondrial membrane potential collapse in the human dermal fibroblast primary cells. Iran. J. Pharm. Res.

